# The Sphere of Institutional Treatment

**Published:** 1913-01-25

**Authors:** 


### II. THE SPHERE OF INSTITUTIONAL TREATMENT.

### The Collier in his Brick Box.

BY A MEDICAL SUPERINTENDENT.

LAST week under this heading we took as our *Orientirung* a concise but frank account of the actual state of general medical practice among a large section of the working-class; showed how it resolved itself into supplying "what the public wants"—that is, plenty of drugs-and-water; how it had brought considered attention to sickness down to the irreducible minimum; how it immediately fell back upon the voluntary hospitals when anything serious had to be dealt with; how it tended to degrade, and physically and professionally to disable, those who undertook it.

### FROM HOME TO INSTITUTION.

Now this—or any—collection of existent evils, it is at once admitted, there is little use in touching briefly in an informative article unless some remedy is indicated as well. With equal readiness, too, we grant that all such consequents must be in close and not easily dissoluble connection with well-established antecedents. We have no sovereign remedy to suggest: the practical spirit pervading this Journal should prevent any suspicion of attempts at *doctrinaire* reform. What we have to ventilate is nothing reactionary, nothing grounded only on theory, but something to trace which endeavour has previously been made in these columns, and that is the noticeable tendency of latter-day civilisation to transfer increasingly the scene of medical attendance from the individual home (particularly the working-class home, with all its lack of convenience) to the institution. To get done with admissions, it should be further allowed that we, over this matter, are in the position of Molière's character. We are jewellers ourselves; or at least the conductors of a jeweller's paper. But in order that none of the arguments in favour of leather may be omitted, it is necessary to get the views of the shoemaker. It is not the judge who states the case for a certain view, but the advocate. Let us hope that a few of the many good reasons which have led in the last two decades to the activity of the Metropolitan Asylums Board, to the founding of sanatoriums, cottage hospitals, works hospitals, and so forth, may find details confirmatory in what follows.

In the first place note carefully that the evils of colliery practice are no result of any want of that much-invoked element—democratic control. In mining villages there are no Lady Bountifuls, no squires, and no parsons with any authority worth talking about. Things are managed by the miners' representatives, and these, although secretly they may hold to the opinions on undiluted democracy of Ibsen's Dr. Stockmann, never, since heroes are scarce, seem to show his courage and out-spokenness. The pit doctor is, to put it less bluntly than the gentleman quoted last week, one of the courtiers of King Demos. And the pathetic thing is that it is just when this monarch, like all other absolute and not very wise ones, feels with pride and satisfaction he has got his own way altogether, that in reality matters are in a bad way with him. He can make the man he pays feel what it is to be a dependant, and can screw medicine out of him instead of taking advice to open bedroom windows or drink less beer. But, after all, there are not so many who will stick to the post of hack and bottle-washer; and those who do so, recoup themselves by taking many more patients than they can see to properly, by retreating to a club in the evenings to get out of the way of needless calls, by physicking symptoms instead of investigating causes, and thus generally cheating their tyrant.

### THE INSTITUTIONAL SPIRIT AT WORK.

It is, indeed, this very feature of excessive democratic control that suggests a remedy. We venture to think the chastening influence exerted on inmates and subjects by a well-ordered institution and system would do something to counteract the unfortunate consequences of letting the many-headed arrange its own doctoring. The Englishman's home is his castle, and the collier in his brick box may appear to listen to advice tendered him; but unless that advice be congenial, or unless he be a man of unusual intelligence and self-control, or unless his medical attendant possess unusual force of character, the advice is not likely to be followed—even if, indeed, brick-box conditions of life permit of it being followed. But introduce him to the mysteries of clean sheets, fresh air, orderly and gentle surroundings, or let him experience the effect on his over-taxed domestic economy of relieving it temporarily of a fretful feverish child, who must be isolated to see if a rash is going to come out, or of a wife in labour, and you will make him a reasonable, enlightened person in matters medical at once. He may come to take as much pride in the *x-ray* installation, a share in which belongs to him, as he formerly did in his right to prefer an unmannerly complaint of the time his old doctor took before paying the sixty-seventh and final visit of a daily round.

[*To be concluded.*]

